# The Kaniadakis Distribution for the Analysis of Income and Wealth Data

**DOI:** 10.3390/e25081141

**Published:** 2023-07-30

**Authors:** Fabio Clementi

**Affiliations:** Department of Political Science, Communication and International Relations, University of Macerata, Via Don Minzoni 22/A, 62100 Macerata, Italy; fabio.clementi@unimc.it; Tel.: +39-0733-258-2560

**Keywords:** income and wealth distribution, parametric modeling, *κ*-generalized model

## Abstract

The paper reviews the “κ-generalized distribution”, a statistical model for the analysis of income data. Basic analytical properties, interrelationships with other distributions, and standard measures of inequality such as the Gini index and the Lorenz curve are covered. An extension of the basic model that best fits wealth data is also discussed. The new and old empirical evidence presented in the article shows that the κ-generalized model of income/wealth is often in very good agreement with the observed data.

## 1. Introduction

The past two decades have seen a resurgence of interest in the study of income and wealth distribution in both the physics [1,2,3,4] and economics [5,6,7,8,9] communities. Scholars have focused particularly on the empirical analysis of large data sets to infer the shape of income and wealth distributions and to develop theoretical models that can reproduce them.

Pareto’s observation that the number of people in a population whose income exceeds *x* is often well approximated by $Cx^{-\alpha }$ was a natural starting point for this field of analysis [10,11,12,13]. However, empirical research has shown that the Pareto distribution accurately models only high income levels, while it does a poor job of describing the lower end of distributions.

As research has continued, new models have been proposed to better describe the data, using either a combination of known statistical distributions [14,15,16,17,18,19,20,21,22] or parametric functional forms for the distribution as a whole. Among these, the two-parameter lognormal [23] and gamma [24] distributions were proposed as models for the size distributions of income and wealth, but later evidence showed that these models tend to exaggerate skewness and perform poorly at the upper end of the empirical distributions [25,26,27,28]. Three-parameter models such as the generalized gamma [29,30,31,32], Singh–Maddala [33], and Dagum Type I [34] provide better fits. These models converge to the Pareto model for large values of income/wealth and accurately describe lower and middle ranges.

Finally, models with more than three parameters have also been suggested to fit income and wealth data. For example, the generalized beta distribution of the second kind (GB2) is a four-parameter distribution that was first described by [35]. It fits the data very well and also includes some of the two- and three-parameter models mentioned above as special or limiting cases. (The generalized beta distribution of the first kind (GB1) [35] and the double Pareto-lognormal distribution [36] are other four-parameter models that fit the data well. Ref. [37] also developed the five-parameter generalized beta distribution family, which includes the GB1 and GB2 as special cases and all of the two- and three-parameter distributions nested inside them. In turn, the double Pareto-lognormal distribution has been generalized into a five-parameter family of distributions called the generalized double Pareto-lognormal distribution [38]. However, closed-form expressions for probability density and/or cumulative distribution functions do not always exist for these “super” models, making fitting them to data computationally difficult and slow due to the need to use numerical methods [39,40]).

Among models that seek to provide a unified framework for describing real-world data, including the power-law tails found in empirical distributions of income and wealth, the κ-generalized distribution has demonstrated exceptional performance and is often seen as a better alternative to other widely used parametric models. This model, which was initially introduced in 2007 and progressively expanded in the years that followed, has its origins in the framework of κ-generalized statistical mechanics [41,42,43,44,45,46]. It has a bulk very similar to the Weibull distribution and an upper tail that decays according to a Pareto power law for high values of income and wealth, providing a sort of middle ground between the two descriptions.

The purpose of this paper is to provide a comprehensive overview of the important results concerning the κ-generalized distribution. The desire to celebrate the 20th anniversary of Kaniadakis’ notable contribution and the belief that an interdisciplinary approach integrating statistical mechanics and economics may give novel insights into economic relationships motivated this work. Giorgio Kaniadakis played a pivotal role in the development of the κ-generalized model, making valuable and direct contributions to its conception. The intention behind presenting information on the fundamental statistical properties and empirical plausibility of this distribution is to convince the reader of its importance and usefulness for future exploration.

The paper is structured as follows. Section 2 introduces the κ-generalized model, covering topics such as interrelations with other distributions, basic statistical properties, and inferential aspects. Section 3 presents recent results of fitting the κ-generalized distribution to empirical income data corresponding to the distribution of household incomes in Greece, and compares the relative merits of alternative income size distribution models using the same data. Section 4 reviews empirical applications showing that the κ-generalized model is often in excellent agreement with observed income data; the κ-generalized mixture model for net worth distribution, which best fits wealth data, is also discussed in this section. Section 5 concludes the paper with some remarks.

## 2. The κ-Generalized Model for Income Distribution

The κ-generalized statistical model, named after [47], is based on the use of κ-deformed exponential and logarithmic functions introduced by Kaniadakis [41,42,43] in the context of special relativity. Within this framework, the ordinary exponential function $\exp\left(x\right)$ deforms into the generalized exponential function $\exp_{\kappa }\left(x\right)$ given by:

(1) $$\exp_{\kappa }\left(x\right)={\left(\sqrt{1+\kappa^{2}x^{2}}+\kappa x\right)}^{\frac{1}{\kappa }},\;x\in R,\;\kappa \in \left[0,1\right).$$

The deformed logarithmic function $\ln_{\kappa }\left(x\right)$, which is defined as the inverse of (1), can be written as:

(2) $$\ln_{\kappa }\left(x\right)=\frac{x^{\kappa }-x^{-\kappa }}{2\kappa },\;\;\;x\in R_{+}.$$

Kaniadakis’ deformed functions have also been successfully used to analyze nonphysical systems. In economics, the κ-deformation has been used to study differentiated product markets [48,49], finance [50,51,52,53,54,55], and the distribution of income by size [47,56,57,58,59,60,61,62]. In the latter case, it is interesting to use such deformed functions because they can be used to statistically describe the entire spectrum of incomes, from the low to the middle range and up to the Pareto tail.

### 2.1. Definitions and Basic Properties

A random variable *X* is said to have a κ-generalized distribution, and we write $X∼\kappa -\mathrm{gen}\left(\alpha ,\beta ,\kappa \right)$, if it has a probability density function (PDF) given by:

(3) $$f\left(x;\alpha ,\beta ,\kappa \right)=\frac{\alpha }{\beta }{\left(\frac{x}{\beta }\right)}^{\alpha -1}\frac{\exp_{\kappa }\left[-{\left(x/\beta \right)}^{\alpha }\right]}{\sqrt{1+\kappa^{2}{\left(x/\beta \right)}^{2\alpha }}},\;x>0,\;\alpha ,\beta >0,\;\kappa \in \left[0,1\right).$$

Its cumulative distribution function (CDF) can be expressed as:

(4) $$F\left(x;\alpha ,\beta ,\kappa \right)=1-\exp_{\kappa }\left[-{\left(x/\beta \right)}^{\alpha }\right].$$

(For a complete description of the κ-generalized distributional properties, the reader is referred to [60] and the references cited therein. A heuristic derivation of the κ-generalized density, showing how this probability distribution emerges naturally within the field of κ-deformed analysis, is given in [61,63]).

**Figure 1:**
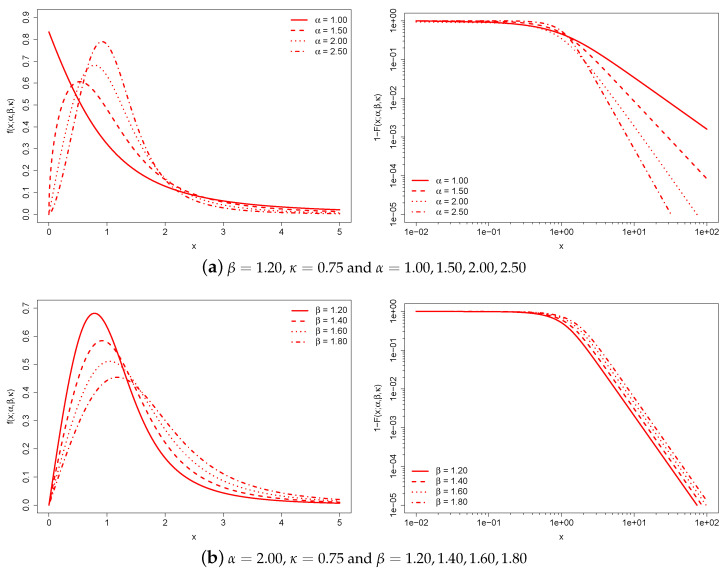

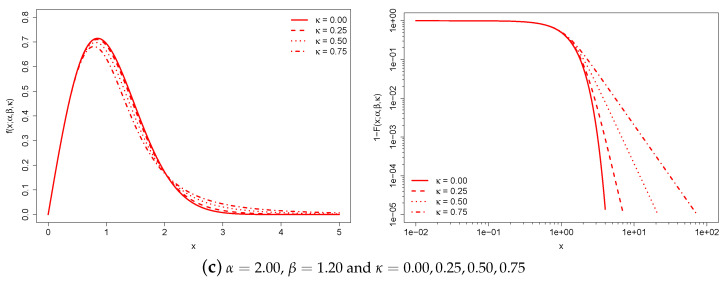
κ-generalized PDF (**left**) and complementary CDF (**right**) for different values of the parameters. The complementary CDF is plotted on double-log axes, which is the standard way to emphasize the right-tail behavior of a distribution.

Figure 1 illustrates the behavior of the κ-generalized PDF and the complementary CDF, $1-F\left(x;\alpha ,\beta ,\kappa \right)$, for various parameter values.

Each of the three graph pairs holds two parameters constant and varies the remaining one.

The constant β is a characteristic scale that has the same dimension as income. For this reason, it takes into account the monetary unit and can be used to adjust for inflation and facilitate cross-country comparisons of income distributions expressed in different monetary units. Increases in the monetary unit result in a global increase in individual income and average income.

The α and κ parameters are scale-free parameters that affect the distribution’s shape. The region around the origin of the κ-generalized distribution is dominated by α, while the upper tail is dominated by both α and κ. Increasing κ leads to a thicker upper tail, while increasing α tapers both tails and increases the concentration of probability mass around the peak of the distribution.

As κ approaches 0, the distribution converges to the Weibull distribution; it is easy to verify that:

(5) $$\lim_{\kappa \to 0}f\left(x;\alpha ,\beta ,\kappa \right)=\frac{\alpha }{\beta }{\left(\frac{x}{\beta }\right)}^{\alpha -1}\exp\left[-{\left(x/\beta \right)}^{\alpha }\right]$$

and:

(6) $$\lim_{\kappa \to 0}F\left(x;\alpha ,\beta ,\kappa \right)=1-\exp\left[-{\left(x/\beta \right)}^{\alpha }\right].$$

(The Weibull distribution is primarily studied in the engineering literature. In physics, it is known as the stretched exponential distribution when α<1. In economics, it has potential for income data, although it has only been used sporadically—some applications can be found in Refs. [29,35,64,65,66,67,68,69].) The distribution behaves similarly to the Weibull model for $x\to 0^{+}$, while for large *x* it approaches a Pareto distribution of the first kind with scale $k=\beta {\left(2\kappa \right)}^{-\frac{1}{\alpha }}$ and shape $a=\frac{\alpha }{\kappa }$, i.e.:

(7) $$f\left(x;\alpha ,\beta ,\kappa \right)\underset{x\to +\infty }{∼}\frac{ak^{a}}{x^{a+1}}$$

and:

(8) $$F\left(x;\alpha ,\beta ,\kappa \right)\underset{x\to +\infty }{∼}1-{\left(\frac{k}{x}\right)}^{a},$$

thus satisfying the weak Pareto law [70]. (Additional versions of the Pareto law were introduced by [71], $\lim_{x\to +\infty }\frac{xf\left(x\right)}{1-F\left(x\right)}=a$, and [30], $\lim_{x\to +\infty }\left[1+\frac{xf^{\prime }\left(x\right)}{f\left(x\right)}\right]=-a$. Since we have: $\lim_{x\to +\infty }\frac{xf\left(x;\alpha ,\beta ,\kappa \right)}{1-F\left(x;\alpha ,\beta ,\kappa \right)}=\frac{\alpha }{\kappa }=a$ and $\lim_{x\to +\infty }\left[1+\frac{xf^{\prime }\left(x;\alpha ,\beta ,\kappa \right)}{f\left(x;\alpha ,\beta ,\kappa \right)}\right]=-\frac{\alpha }{\kappa }=-a$, the κ-generalized distribution also obeys these alternative versions of the weak Pareto law.)

Equation (4) implies that the quantile function is available in closed form:

(9) $$F^{-1}\left(u;\alpha ,\beta ,\kappa \right)=x_{u}=\beta {\left[\ln_{\kappa }\left(\frac{1}{1-u}\right)\right]}^{\frac{1}{\alpha }},\;0<u<1,$$

an attractive feature for generating random numbers from a κ-generalized distribution. The median of the distribution is:

(10) $$x_{\mathrm{med}}=\beta {\left[\ln_{\kappa }\left(2\right)\right]}^{\frac{1}{\alpha }},$$

and the mode occurs at:

(11) $$x_{\mathrm{mode}}=\beta {\left[\frac{\alpha^{2}+2\kappa^{2}\left(\alpha -1\right)}{2\kappa^{2}\left(\alpha^{2}-\kappa^{2}\right)}\right]}^{\frac{1}{2\alpha }}{\left\{\sqrt{1+\frac{4\kappa^{2}\left(\alpha^{2}-\kappa^{2}\right){\left(\alpha -1\right)}^{2}}{{\left[\alpha^{2}+2\kappa^{2}\left(\alpha -1\right)\right]}^{2}}}-1\right\}}^{\frac{1}{2\alpha }}$$

if α>1; otherwise, the distribution is zero-modal with a pole at the origin.

Finally, the *r*th raw moment of the κ-generalized distribution is equal to:

(12) $$\langle x^{r}\rangle =\int_{0}^{\infty }x^{r}f\left(x;\alpha ,\beta ,\kappa \right)dx=\beta^{r}{\left(2\kappa \right)}^{-\frac{r}{\alpha }}\frac{\Gamma \left(1+\frac{r}{\alpha }\right)}{1+\frac{r}{\alpha }\kappa }\frac{\Gamma \left(\frac{1}{2\kappa }-\frac{r}{2\alpha }\right)}{\Gamma \left(\frac{1}{2\kappa }+\frac{r}{2\alpha }\right)},$$

where $\Gamma \left(\cdot \right)$ denotes the gamma function, and exists for $-\alpha <r<\frac{\alpha }{\kappa }$. Specifically:

(13) $$\langle x\rangle =\beta {\left(2\kappa \right)}^{-\frac{1}{\alpha }}\frac{\Gamma \left(1+\frac{1}{\alpha }\right)}{1+\frac{1}{\alpha }\kappa }\frac{\Gamma \left(\frac{1}{2\kappa }-\frac{1}{2\alpha }\right)}{\Gamma \left(\frac{1}{2\kappa }+\frac{1}{2\alpha }\right)}$$

is the mean of the distribution and:

(14) $$\langle x^{2}\rangle -{\langle x\rangle }^{2}=\beta^{2}{\left(2\kappa \right)}^{-\frac{2}{\alpha }}\left\{\frac{\Gamma \left(1+\frac{2}{\alpha }\right)}{1+2\frac{\kappa }{\alpha }}\frac{\Gamma \left(\frac{1}{2\kappa }-\frac{1}{\alpha }\right)}{\Gamma \left(\frac{1}{2\kappa }+\frac{1}{\alpha }\right)}-{\left[\frac{\Gamma \left(1+\frac{1}{\alpha }\right)}{1+\frac{\kappa }{\alpha }}\frac{\Gamma \left(\frac{1}{2\kappa }-\frac{1}{2\alpha }\right)}{\Gamma \left(\frac{1}{2\kappa }+\frac{1}{2\alpha }\right)}\right]}^{2}\right\}$$

is the variance.

### 2.2. Measuring Income Inequality Using the κ-Generalized Distribution

The concept of inequality in economics dates back to Pareto’s early work [10,11,12,13], which showed that the top 20% of population held about 80% of total income/wealth. Later, the American economist Lorenz [72] introduced the Lorenz curve, a widely used tool for measuring income/wealth inequality. This curve measures the difference in income or wealth distribution from an equal distribution. If there is perfect equality, the Lorenz curve coincides with the diagonal of a unit square, while worsening distribution (more inequality) moves the curve away from the diagonal.

The Lorenz curve for a random variable *X* with CDF $F\left(x\right)$ and finite mean $\langle x\rangle =\int xdF\left(x\right)$ is defined as [73]:

(15) $$L\left(u\right)=\frac{1}{\langle x\rangle }\int_{0}^{u}F^{-1}\left(t\right)dt,\;u\in \left[0,1\right].$$

Using the closed form of the quantile function $F^{-1}\left(u\right)$ of the κ-generalized distribution, the Lorenz curve can be reformulated as follows [74]:

(16) $$L\left(u\right)=I_{x}\left(1+\frac{1}{\alpha },\frac{1}{2\kappa }-\frac{1}{2\alpha }\right),\;\;\;x=1-{\left(1-u\right)}^{2\kappa },$$

where $I_{x}\left(\cdot ,\cdot \right)$ is the regularized incomplete beta function defined in terms of the incomplete beta function and the complete beta function, that is, $I_{x}\left(\cdot ,\cdot \right)=\frac{B_{x}\left(\cdot ,\cdot \right)}{B\left(\cdot ,\cdot \right)}$. The curve (16) exists if and only if $\frac{\alpha }{\kappa }>1$. In particular, if $X_{i}∼\kappa -\mathrm{gen}\left(\alpha_{i},\beta_{i},\kappa_{i}\right)$, i=1,2, the necessary and sufficient conditions for the Lorenz curves of $X_{1}$ and $X_{2}$ not to intersect (otherwise, it would be impossible to determine which distribution has more inequality) are [58]:

(17) $$\alpha_{1}\geq \alpha_{2}\;\mathrm{and}\;\frac{\alpha_{1}}{\kappa_{1}}\geq \frac{\alpha_{2}}{\kappa_{2}}.$$

**Figure 2:**
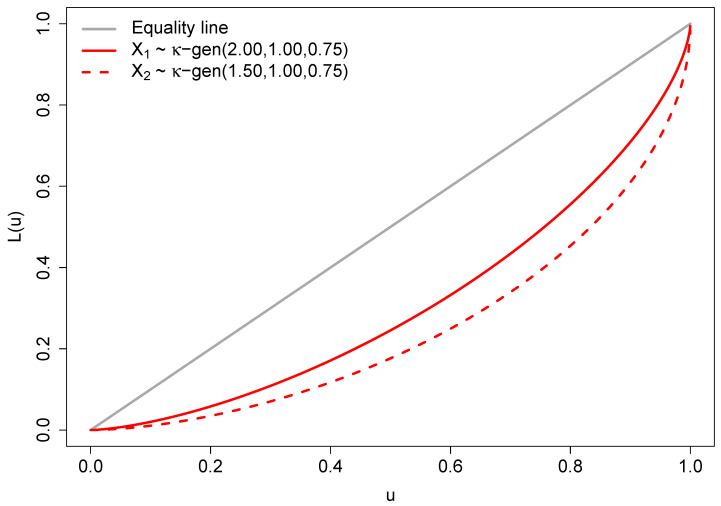
Lorenz curves for two κ-generalized distributions.

The Lorenz curves of two κ-generalized distributions $X_{1}$ and $X_{2}$ with parameters chosen according to (17) are illustrated in Figure 2. The depicted curves indicate that $X_{1}$ exhibits lower inequality compared to $X_{2}$, as the Lorenz curve of $X_{1}$ does not intersect or fall below that of $X_{2}$.

Economists have employed statistical metrics to quantify income and wealth inequality. The Gini coefficient, developed in 1914 by the Italian statistician Gini [75], is one of the best known. From the general definition $G=1-\frac{1}{\langle x\rangle }\int_{0}^{\infty }{\left[1-F\left(x\right)\right]}^{2}dx$ due to [76], the Gini coefficient associated with the κ-generalized distribution is:

(18) $$G=1-\frac{2\alpha +2\kappa }{2\alpha +\kappa }\frac{\Gamma \left(\frac{1}{\kappa }-\frac{1}{2\alpha }\right)}{\Gamma \left(\frac{1}{\kappa }+\frac{1}{2\alpha }\right)}\frac{\Gamma \left(\frac{1}{2\kappa }+\frac{1}{2\alpha }\right)}{\Gamma \left(\frac{1}{2\kappa }-\frac{1}{2\alpha }\right)}.$$

Using the Stirling approximation for the gamma function, $\Gamma \left(z\right)\approx \sqrt{2\pi }z^{z-\frac{1}{2}}\exp\left(-z\right)$, and taking the limit as κ→0 in Equation (18), after some simplification one arrives at $G=1-2^{-\frac{1}{\alpha }}$, which is the explicit form of the Gini coefficient for the Weibull distribution (see e.g., [77], p. 177). Since the exponential distribution is a special case of the Weibull distribution with a shape parameter of 1, it follows directly that for κ→0 and α=1, the exponential law is also a special limiting case of the κ-generalized distribution with a true Gini coefficient of one half [16].

The Gini coefficient is a widely used measure of inequality, but it makes specific assumptions about income differences in different parts of the distribution. It is most sensitive to transfers around the middle of the income distribution and least sensitive to transfers among the very rich or very poor [78]. Differently, the generalized entropy class of inequality measures [79,80,81,82,83] provides a range of bottom-to-top sensitive indices used by analysts to assess inequality in different parts of the income distribution. The expression for this class of inequality indices in terms of the κ-generalized parameters is [57]:

(19) $$GE\left(\theta \right)=\frac{1}{\theta^{2}-\theta }\left\{{\left(\frac{\beta }{m}\right)}^{\theta }\left[\frac{{\left(2\kappa \right)}^{-\frac{\theta }{\alpha }}}{1+\frac{\theta }{\alpha }\kappa }\frac{\Gamma \left(\frac{1}{2\kappa }-\frac{\theta }{2\alpha }\right)}{\Gamma \left(\frac{1}{2\kappa }+\frac{\theta }{2\alpha }\right)}\Gamma \left(1+\frac{\theta }{\alpha }\right)\right]-1\right\},\;\theta \neq 0,1,$$

where $m=\langle x\rangle$ denotes the mean of the distribution given by Equation (13). Formula (19) defines a class because $GE\left(\theta \right)$ takes different forms depending on the value given to θ, the parameter that describes the sensitivity of the index to income differences in different parts of the income distribution—the more positive or negative θ is, the more sensitive $GE\left(\theta \right)$ is to income differences at the top or bottom of the distribution. Two limiting cases of (19), obtained when the parameter θ is set to 0 and 1, have gained attention in practical work for the purpose of measuring inequality; these are the mean logarithmic deviation index:

(20) $$MLD=\lim_{\theta \to 0}GE\left(\theta \right)=\frac{1}{\alpha }\left[\gamma +\psi \left(\frac{1}{2\kappa }\right)+\ln\left(2\kappa \right)-\alpha \ln\left(\frac{\beta }{m}\right)+\kappa \right],$$

where $\gamma =-\psi \left(1\right)$ is the Euler–Mascheroni constant and $\psi \left(z\right)=\Gamma^{{}^{\prime }}\left(z\right)/\Gamma \left(z\right)$ is the digamma function, and the Theil index [84]:

(21) $$\begin{array}{cc} T=\lim_{\theta \to 1}GE\left(\theta \right)= & \frac{1}{\alpha }\left[\psi \left(1+\frac{1}{\alpha }\right)-\frac{1}{2}\psi \left(\frac{1}{2\kappa }-\frac{1}{2\alpha }\right)-\frac{1}{2}\psi \left(\frac{1}{2\kappa }+\frac{1}{2\alpha }\right)\right.\\ \; & \left.-\ln\left(2\kappa \right)+\alpha \ln\left(\frac{\beta }{m}\right)-\frac{\alpha \kappa }{\alpha +\kappa }\right], \end{array}$$

where the former is more sensitive to variations in the lower tail, while the latter is more sensitive to variations in the upper tail [85]. (Equation (19) is not defined for θ=0 and θ=1, as $\left(\theta^{2}-\theta \right)=0$ in both cases. Expressions for these values of θ are therefore derived using l’Hôpital rule, which allows evaluating limits of indeterminate forms using derivatives. Expressions for any $GE\left(\theta \right)$ index other than the cases θ=0,1 can be derived by simple substitution—see for example [60]).

Finally, the class of inequality measures introduced by Atkinson [86] can be derived from (19) by exploiting the relationship [87,88]:

(22) $$A\left(\epsilon \right)=1-{\left[\epsilon \left(\epsilon -1\right)GE\left(1-\epsilon \right)+1\right]}^{\frac{1}{1-\epsilon }},\;\epsilon >0,\;\epsilon \neq 1,$$

where ϵ=1−θ is the inequality aversion parameter. As ϵ increases, $A\left(\epsilon \right)$ becomes more sensitive to transfers among lower incomes and less sensitive to transfers among top incomes [78]. The limiting form of (22) is $A\left(1\right)=1-\exp\left(-MLD\right)$. (All measures considered here are functions of distributional moments, whose existence depends on conditions assuring the convergence of the appropriate integrals. The Gini coefficient (18) exists if and only if the mean of the distribution $\langle x\rangle =\int_{0}^{\infty }xf\left(x;\alpha ,\beta ,\kappa \right)dx$ converges, which is true if and only if $\frac{\alpha }{\kappa }>1$. According to [89], parametric income distribution models share the existence problem of popular inequality measures).

### 2.3. Estimation

The κ-generalized distribution’s parameters can be estimated using the maximum likelihood technique, which produces estimators with good statistical properties [90,91]. If sample observations $x=\left\{x_{1},\ldots ,x_{n},\right\}$ are independent, the likelihood function is as follows:

(23) $$L\left(x;\theta \right)=\prod_{i=1}^{n}f{\left(x_{i};\theta \right)}^{w_{i}}=\prod_{i=1}^{n}{\left\{\frac{\alpha }{\beta }{\left(\frac{x_{i}}{\beta }\right)}^{\alpha -1}\frac{\exp_{\kappa }\left[-{\left(x_{i}/\beta \right)}^{\alpha }\right]}{\sqrt{1+\kappa^{2}{\left(x_{i}/\beta \right)}^{2\alpha }}}\right\}}^{w_{i}},$$

where $f\left(x_{i};\theta \right)$ denotes the PDF, $\theta =\left\{\alpha ,\beta ,\kappa \right\}$ the vector of unknown parameters, $w_{i}$ the weight of the *i*th observation, and *n* the sample size. This leads to the problem of solving the partial derivatives with respect to α, β and κ for the log-likelihood function:

(24) $$l\left(x;\theta \right)=\ln\left[L\left(x;\theta \right)\right]=\sum_{i=1}^{n}w_{i}\ln\left[f\left(x_{i};\theta \right)\right],$$

which is the same as finding the solution to the following nonlinear system of equations:

(25) $$\sum_{i=1}^{n}w_{i}\frac{\partial }{\partial \alpha }\ln\left[f\left(x_{i};\theta \right)\right]=0,$$

(26) $$\sum_{i=1}^{n}w_{i}\frac{\partial }{\partial \beta }\ln\left[f\left(x_{i};\theta \right)\right]=0,$$

(27) $$\sum_{i=1}^{n}w_{i}\frac{\partial }{\partial \kappa }\ln\left[f\left(x_{i};\theta \right)\right]=0.$$

However, the derivation of explicit expressions for maximum likelihood estimators of the three κ-generalized parameters poses a challenge due to the absence of feasible analytical solutions. The utilization of numerical optimization algorithms becomes therefore imperative in order to solve the maximum likelihood estimation problem.

## 3. Application to the Income Distribution in Greece

To celebrate 20 years of Kaniadakis’ contribution, it seems appropriate to consider the income distribution in his native Greece to demonstrate the κ-generalized model’s capacity to fit real-world data. First, income data for parameter estimation are briefly described. Next, the κ-generalized distribution is fitted to Greek household income data. Finally, using the same income microdata, different income size distribution models are compared.

### 3.1. Description of the Income Data

Income distribution data for Greece were obtained from the Luxembourg Income Study (LIS) database, which provides public access to household-level data files for various countries, including both developed and developing economies. The data are remote-accessible, requiring program code to be sent to LIS rather than being run directly by the user. At the time of writing, LIS contains Greek income distribution data for the following years: 1995, 2000, 2004, 2007, 2010, 2013, and 2016. The data set used for this review is the 2016 data set based on the 2017 wave of the Greek EU-SILC survey conducted by the Hellenic Statistical Authority (ELSTAT). (EU-SILC is a cross-sectional and longitudinal sample survey coordinated by Eurostat, focusing on income, poverty, social exclusion, and living conditions in the European Union.) The sample size is 22,555 households.

The definition of income is “household disposable income”, which is the income available to households to support consumption expenditure and saving during the reference period. The measure includes income from work, wealth, and direct government benefits, but subtracts direct taxes paid. It does not include sales taxes or noncash benefits, such as healthcare provided by a government or employer. Additionally, the income definition excludes income from capital gains, a significant source of nonwage income for wealthy individuals. As a result, many top incomes are likely to be underestimated.

Household disposable income is expressed in euro and “equivalized”, i.e., divided by the square root of household size to adjust for differences in household demographics. Prior to equivalization, top and bottom coding is applied to set limits for extreme values. We also exclude all households with missing disposable income and use person-adjusted weights (the product of the household weights and the number of household members) when generating income indicators for the total population and estimating model parameters.

### 3.2. Results of Fitting

**Figure 3:**
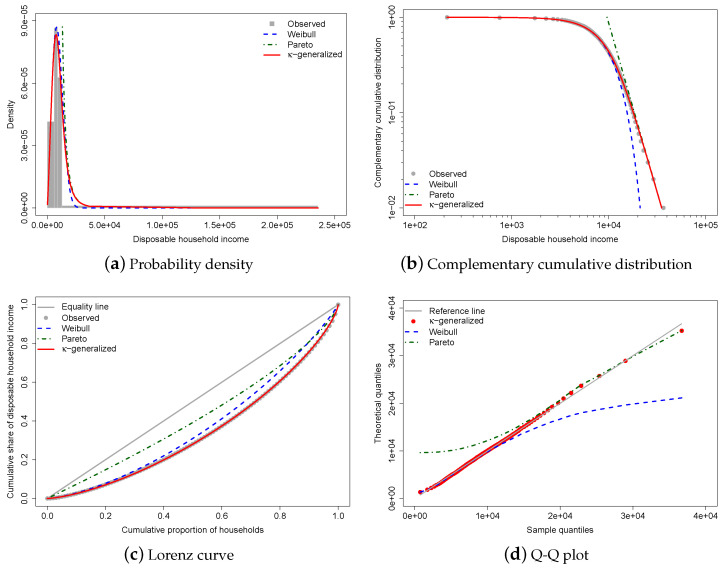
κ-generalized distribution fitted to Greek household income data for 2016. The red solid line represents the κ-generalized model, which fits the data well over the whole range from low to high incomes, including the middle income region. It is compared to the Weibull (blue dashed line) and Pareto power-law (green dashed line) distributions. The complementary cumulative distribution is plotted on double-log axes, emphasizing the right-tail behavior of the distribution. The Lorenz curve plot compares the empirical and theoretical curves, with the gray solid line representing the Lorenz curve of a society with equal income distribution. The Q-Q plot of sample percentiles versus theoretical percentiles of the fitted κ-generalized shows excellent fit, with corresponding percentiles being close to the $45^{\circ }$ line from the origin.

Figure 3 shows the results of fitting the κ-generalized distribution to empirical income data corresponding to the distribution of household income in Greece for the year 2016.

The best-fitting parameter values were determined using maximum likelihood estimation, resulting in estimates of α=2.233±0.017, β=10,667±46, and κ=0.630±0.014. The small errors indicate accurate estimations, and the comparison between the observed and fitted probabilities in panels (a) and (b) of Figure 3 suggests that the κ-generalized distribution has great potential for describing data across the range of low-to-middle-income to high-income power-law regimes, including the intermediate region where Weibull and Pareto distributions show clear departures. (In Figure 3, the curves for the Pareto and Weibull distributions have been drawn by expressing their parameters in terms of the estimated κ-generalized parameters—see Section 2.1).

Panel (c) of the same figure displays data points for the empirical Lorenz curve superimposed on the theoretical curve given by Equation (16) with estimates replacing α and κ as necessary. This formula, represented by the red solid line in the plot, matches the data exceptionally well. In addition, the plot contrasts the empirical Lorenz curve with the theoretical curves associated with the Weibull and Pareto distributions, respectively, given by:

(28) $$\lim_{\kappa \to 0}L\left(u\right)=P\left(1+\frac{1}{\alpha },-\ln\left(1-u\right)\right),$$

where $P\left(\cdot ,\cdot \right)$ is the lower regularized incomplete gamma function, and:

(29) $$\lim_{x\to \infty }L\left(u\right)=1-{\left(1-u\right)}^{1-\frac{1}{a}}.$$

As one can easily see, these curves tell only a small part of the story.

To provide an indirect check on the validity of the parameter estimation, we have also computed predicted values for median and mean household disposable income, as well as the Gini and Atkinson coefficients—the latter with the inequality aversion parameter ϵ equal to 1. The results, obtained by substituting the estimated parameters into relevant expressions, are presented in Table 1, along with their empirical counterparts, corresponding to the LIS staff’s “Inequality and Poverty Key Figures” for the considered country and year. (In this article, inequality measures are calculated using the most recent version of DASP, the Distributive Analysis Stata Package [92], which is available at http://dasp.ecn.ulaval.ca/—accessed on 26 June 2023. The complete set of corresponding “key figures” is available in an Excel workbook that can be downloaded from https://www.lisdatacenter.org/data-access/key-figures/—accessed on 26 June 2023).

The κ-generalized distribution predictions are fully covered by asymptotic normal 95% confidence intervals, confirming excellent agreement between the model and sample observations.

The linear behavior of the quantile-quantile (Q-Q) plot of sample percentiles against the fitted κ-generalized distribution and its limiting cases, shown in panel (d) of Figure 3, confirms the model’s validity as well as the fact that the Weibull and Pareto distributions provide partial and incomplete data descriptions.

### 3.3. Comparisons of Alternative Distributions

This section compares the κ-generalized distribution’s performance with other parametric models, including the three-parameter generalized gamma [93], Singh–Maddala [33], and Dagum type I [34] distributions, which have the following PDFs, respectively:

(30) $$f\left(x;a,\beta ,p\right)=\frac{ax^{ap-1}\exp\left[-{\left(x/\beta \right)}^{a}\right]}{\beta^{ap}\Gamma \left(a\right)},\;x>0,\;a,\beta ,p>0,$$

(31) $$f\left(x;a,b,q\right)=\frac{aqx^{a-1}}{b^{a}{\left[1+{\left(x/b\right)}^{a}\right]}^{1+q}},\;x>0,\;a,b,q>0,$$

(32) $$f\left(x;a,b,p\right)=\frac{apx^{ap-1}}{b^{ap}{\left[1+{\left(x/b\right)}^{a}\right]}^{p+1}},\;x>0,\;a,b,p>0.$$

Ref. [77] provides analytical expressions for distribution functions, moments, and tools for inequality measurement, including the Lorenz curve and Gini coefficient. Refs. [87,94] provide formulas for generalized entropy measures of the GB2 distribution, from which the Singh–Maddala and Dagum versions are easily obtained. For the generalized gamma distribution, closed expressions for the Theil entropy index and the mean logarithmic deviation are given in Refs. [85,95]. (Let *X* be a random variable following the generalized beta distribution of the second kind (GB2) with parameters *a*, *b*, *p*, and *q*, i.e., $X∼\mathrm{GB}2\left(a,b,p,q\right)$. The Singh–Maddala distribution is the special case of the GB2 distribution when p=1; the Dagum type I distribution is the special case when q=1. For a discussion of other special cases, see [35,77]).

Table 2 displays maximum likelihood estimates for the models under consideration.

The κ-generalized model offers the best results, with parameter standard errors derived from the inverse Hessian matrix being the lowest among competing income distribution models.

The root mean square error and mean absolute error between observed and predicted probabilities were used to determine which distribution best fits the data. These goodness-of-fit measures are, respectively, defined by:

(33) $$RMSE=\sqrt{\frac{1}{n}\sum_{i=1}^{n}{\left[{\hat{F}}_{W}\left(t\right)-F\left(x_{i};\hat{\theta }\right)\right]}^{2}}$$

and:

(34) $$MAE=\frac{1}{n}\sum_{i=1}^{n}\left|{\hat{F}}_{W}\left(t\right)-F\left(x_{i};\hat{\theta }\right)\right|,$$

where ${\hat{F}}_{W}\left(t\right)=\frac{1}{W}\sum_{i=1}^{n}w_{i}1\left\{x_{i}\leq t\right\}$, with $W=\sum_{i=1}^{n}w_{i}$, denotes the weighted empirical cumulative distribution function—equal to the sum of the income weights where x≤t divided by the total sum of weights—and $\hat{\theta }$ is the vector of estimated parameters. (In the formulas above, $1\left\{\cdot \right\}$ is an indicator function that takes the value 1 if the condition in $\left\{\cdot \right\}$ is true, 0 otherwise.) The RMSE and MAE between the observed and estimated Lorenz curves have also been used as goodness-of-fit criteria, as they are expected to better reflect the accuracy of the inequality estimates. These additional measures are given by:

(35) $$LRMSE=\sqrt{\frac{1}{n}\sum_{i=1}^{n}{\left[L_{i}-L\left(\lambda_{i};\hat{\theta }\right)\right]}^{2}}$$

and:

(36) $$LMAE=\frac{1}{n}\sum_{i=1}^{n}\left|L_{i}-L\left(\lambda_{i};\hat{\theta }\right)\right|,$$

where $\lambda_{i}={\hat{F}}_{W}\left(t\right)$ and $L_{i}$ denote the cumulative share of population and income, respectively, up to percentile *i*—i.e., $\left(\lambda_{i},L_{i}\right)$ is a point on the empirical Lorenz curve.

Based on the above goodness-of-fit criteria, the κ-generalized model is clearly the best fit. As shown in the last three columns of Table 2, the generalized gamma, Singh–Maddala, and Dagum type I have larger RMSE and MAE values for both probabilities and Lorenz curves, suggesting that these models perform worse than the κ-generalized distribution.

The performance of the four models is further evaluated by considering the accuracy of selected distributional statistics implied by parameter estimates. Table 3 presents the predicted values for the median, mean, and several inequality measures derived from estimates in Table 2. (The Gini coefficient of the generalized gamma distribution is available in [35] as a long expression involving the Gaussian hypergeometric function ${}_{2}F_{1}$, which is not currently available in the online statistical evaluator provided by the LIS web-based interface. An estimate of the Gini index for the generalized gamma distribution was therefore obtained by numerically integrating the area between the predicted Lorenz curve and the line of hypothetical equality. Ref. [96] reviews various methods for numerically estimating the Gini).

**Figure 4:**
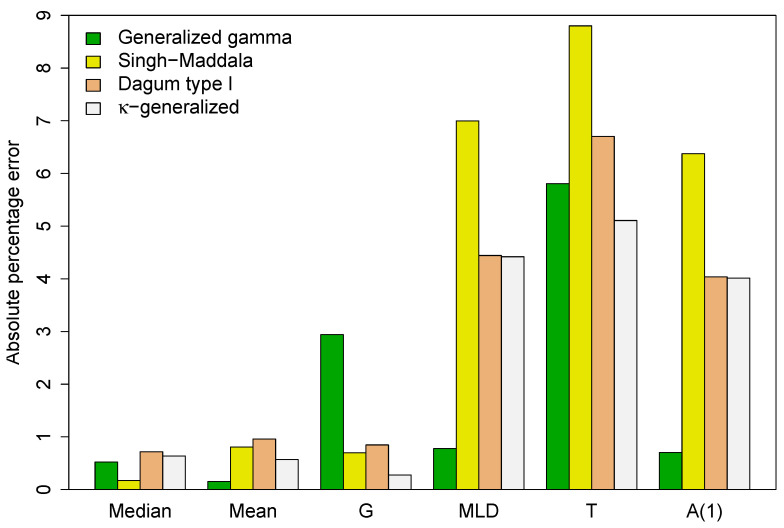
Absolute percentage error between the predicted values for key distributional summary measures and their sample counterparts.

For each of the models examined, the accuracy of the implied statistics is evaluated by calculating the absolute percentage error:

(37) $$APE=\frac{\left|P-A\right|}{A}\times 100$$

between the predicted values (*P*) and the actual sample estimates (*A*) given in Table 3. The results are summarized in Figure 4.

Except for the median, the κ-generalized distribution has more accurate implied estimates of selected distributional statistics than the Singh–Maddala and Dagum type I models, with the Gini coefficient being significantly more accurate. This implies that the κ-generalized estimation procedure preserves the mean characteristic of the analyzed data and accurately models intra- and/or inter-group variation. Additionally, when considering income differences in different parts of the income distribution, the κ-generalized provides more accurate estimates than the two competitors of the MLD index, Theil index *T* and the Atkinson inequality measure $A\left(1\right)$. The Gini is an inequality index sensitive to the middle, while the other indices are more sensitive to the top and bottom of the income distribution. These results support the closest approximation to the income distribution found for the κ-generalized model.

**Figure 5:**
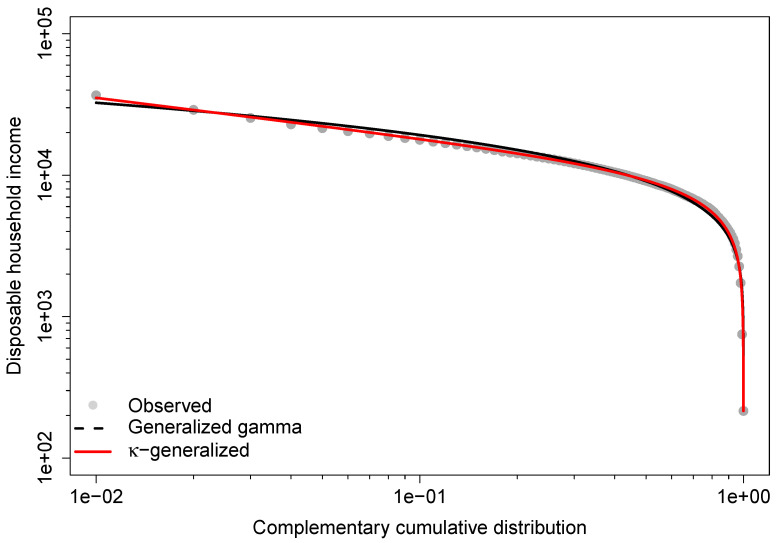
Zipf plot for the 2016 Greek household income data. The lines are the predicted Zipf plots obtained from the fit of the generalized gamma and κ-generalized models.

The κ generalized distribution also outperforms the generalized gamma in predicting the Gini coefficient and Theil index, while the generalized gamma provides more accurate estimates for the MLD index, the $A\left(1\right)$ measure, the median, and the mean. This agreement is due to better fit in the lower part of the observed distribution, while disagreements arise from poorer fit in the upper-middle range, especially at the top end. This is demonstrated by the double-logarithmic plot in Figure 5, known as the Zipf plot, which shows the relationship between income and the complementary CDF of income for the data under study.

The Zipf plot is natural to use when looking at the upper part of the distribution because it puts more emphasis on the upper tail and makes it easier to detect deviations in that part of the distribution from what a model would predict [97]. The lines show the Zipf plots that were predicted by fitting the generalized gamma and κ-generalized models. As the graph shows, both are pretty close to the actual data in the lower part of the income distribution. However, the empirical observations of the upper tail are very different from what the generalized gamma says they should be, while the theoretical Zipf plot for the κ-generalized distribution is much closer to the empirical one in the same part of the observed income distribution.

## 4. Applications of κ-Generalized Models to Income and Wealth Data

Apart from the one considered in this review, there have been numerous applications of the κ-generalized model to real-world income data over the past two decades.

The first study was conducted by [47], who analyzed 2001–2002 household incomes in Germany, Italy, and the United Kingdom. They found excellent agreement between the model and the empirical distributions across the full spectrum of incomes, including the intermediate income range where clear deviation was found when the Weibull model and pure Pareto law were used for interpolation.

The κ-generalized distribution was later applied to Australian household incomes in 2002–2003 [56] and US family incomes in 2003 [56,57]. The model again described the entire income range well and accurately estimated the inequality level in both countries using the Lorenz curve and Gini measure.

Comparative studies that fit multiple distributions to the same data are crucial for comparing performance. For example, Ref. [58], which examined the distribution of household income in Italy from 1989 to 2006, showed that the κ-generalized model outperforms three-parameter competitors such as the Singh–Maddala and Dagum type I distributions, except for the GB2, which has an extra parameter. The model has also also been used to analyze household income data for Germany between 1984 and 2007, the United Kingdom between 1991 and 2004, and the United States between 1980 and 2005. In many cases, the distribution of household income is observed to conform to the κ-generalized model, rather than the Singh–Maddala or Dagum type I distributions. In particular, the κ-generalized distribution is found to outperform competitors in the right tail of the data. The three-parameter κ-generalized model provides superior income inequality estimates even when the fit is worse than distributions belonging to the GB2 family, as obtained by [98] when comparing US and Italian income data for the 2000s. Finally, Ref. [60] finds that the κ-generalized distribution offers a superior fit to the data and, in many cases, estimates income inequality more accurately than alternatives using household income data for 45 countries from Wave IV to Wave IX of the LIS database. (Four-parameter extensions of the κ-generalized distribution, called *extended κ-generalized distributions of the first and second kind*—EκG1 and EκG2, respectively—were introduced by [74]. These two extensions are not discussed here, but Refs. [60,61,74] provide formulas for the moments, Lorenz curve, Gini index, coefficient of variation, mean logarithmic deviation, and Theil index for both the models. The new variants of the κ-generalized distribution outperform other four-parameter models in almost all cases, especially in estimating inequality indices with greater precision. In addition, a κ-deformation of the generalized gamma distribution with a power-law tail has recently been proposed by [99], to which the reader is referred for further details.)

The κ-generalized distribution has also been used to analyze the singularities of survey data on *net* wealth, which is gross wealth minus total debt [60,61,100]. These data show highly significant frequencies of households or individuals with wealth that is either null or negative. The κ-generalized model of wealth distribution is a mixture of an atomic and two continuous distributions. The atomic distribution accounts for economic units with no net worth, while a Weibull function accounts for negative net worth data. Positive net worth values, on the other hand, are represented by the κ-generalized model (3). The κ-generalized mixture model for wealth distribution was used to model US net worth data from 1984 to 2011 [100]. The model was generally accurate and its performance was superior to that of finite mixture models based on the Singh–Maddala and Dagum type I distributions for positive net worth values. Similar results were later obtained by Ref. [60] when analyzing net wealth data for nine countries selected from the Luxembourg Wealth Study (LWS) database. (The Luxembourg Wealth Study database—see https://www.lisdatacenter.org/our-data/lws-database/, accessed on 26 June 2023—is a collaborative project to assemble existing microdata on household wealth into a coherent database, aiming to do for wealth what the LIS database has achieved for income. The LWS was officially launched in 2004 and currently provides wealth data sets for several countries and years).

## 5. Concluding Remarks

The κ-generalized distribution, a statistical model developed over several years of collaborative, multidisciplinary research, is a valuable tool for studying income and wealth distributions. This article discussed its basic properties, relationships with other distributions, and important extensions. It also discussed common inequality measures such as the Lorenz curve and Gini index, and how they can be computed from κ-generalized parameter estimates. A review of empirical applications showed excellent agreement with observed data. It is hoped that the collection of all these results in a single source will facilitate and promote the use of the κ-generalized distribution.

## Figures and Tables

**Table 1:** Observed and predicted values of the median, the mean, the Gini index *G* and the Atkinson inequality measure $A\left(1\right)$.

Statistic	Observed			Predicted
	Value	LB ^a^	UB ^b^	
Median	9123	8983	9264	9181
Mean	10,548	10,292	10,805	10,488
*G*	0.323	0.312	0.334	0.322
$A\left(1\right)$	0.179	0.169	0.189	0.172

Notes: ${}^{a}$ lower bound of the 95% normal-based confidence interval obtained by adding −1.96 times the standard error to the sample indicator; ${}^{b}$ upper bound of the 95% normal-based confidence interval obtained by adding +1.96 times the standard error to the sample indicator. Source: author’s calculations based on Greek LIS data for 2016.

**Table 2:** Maximum likelihood estimates for the generalized gamma, Singh–Maddala, Dagum type I and κ-generalized models of income distribution.

Model ${}^{a}$		Parameters ${}^{b}$				Goodness-of-Fit Criteria ${}^{c,d}$			
		a (α)	b (β)	q, p, κ		RMSE	MAE	LRMSE	LMAE
GG		0.684	829	5.475		2.325	2.047	0.939	0.812
		(0.018)	(115)	(0.270)					
SM		2.441	12,531	1.835		0.716	0.574	0.319	0.187
		(0.021)	(219)	(0.053)					
D		3.705	11,705	0.560		0.539	0.437	0.281	0.211
		(0.041)	(104)	(0.011)					
κ-gen		2.233	10,667	0.630		0.530	0.418	0.188	0.139
		(0.017)	(46)	(0.014)					

Notes: ${}^{a}$ GG = generalized gamma, SM = Singh-Maddala, D = Dagum type I, κ-gen = κ-generalized; ${}^{b}$ numbers in parentheses: estimated standard errors; ${}^{c}$RMSE = root mean square error, MAE = mean absolute error, LRMSE = root mean square error between the observed and estimated Lorenz curves, LMAE = mean absolute error between the observed and estimated Lorenz curves; ${}^{d}$ values multiplied by 100. Source: author’s calculations based on Greek LIS data for 2016.

**Table 3:** Observed and predicted values of selected distributional statistics.

Statistic ^a^		Observed				Predicted ^d^			
		Value	LB ^b^	UB ^c^		GG	SM	D	κ-gen
Median		9123	8983	9264		9076	9108	9189	9181
Mean		10,548	10,292	10,805		10,532	10,463	10,447	10,488
*G*		0.323	0.312	0.334		0.333	0.321	0.320	0.322
MLD		0.197	0.185	0.209		0.196	0.183	0.188	0.188
*T*		0.191	0.175	0.207		0.180	0.174	0.178	0.181
$A\left(1\right)$		0.179	0.169	0.189		0.178	0.168	0.172	0.172

Notes: ${}^{a}$*G* = Gini index, MLD = mean logarithmic deviation index, *T* = Theil index, $A\left(1\right)$ = Atkinson coefficient with inequality aversion parameter ϵ equal to 1; ${}^{b}$ lower bound of the 95% normal-based confidence interval obtained by adding −1.96 times the standard error to the sample indicator; ${}^{c}$ upper bound of the 95% normal-based confidence interval obtained by adding +1.96 times the standard error to the sample indicator; ${}^{d}$ GG = generalized gamma, SM = Singh–Maddala, D = Dagum type I, κ-gen = κ-generalized. Source: author’s calculations based on Greek LIS data for 2016.

## Data Availability

The Luxembourg Income Study Database (https://www.lisdatacenter.org/, accessed on 26 June 2023) provides remote access to the microdata through a web-based Job Submission Interface (LISSY). Users have to register to the platform and submit through the LISSY interface their statistical programs written in R, SAS, SPSS or Stata. Data analysis was performed using Stata software version 17 [101] while graphs were generated using R software version 4.3.1 [102]. To allow reproduction of the analysis, software code used in this article is available from the author on request.
